# Infected urachal cyst in an adult: a case report and review of the literature

**DOI:** 10.4076/1757-1626-2-6422

**Published:** 2009-06-25

**Authors:** Kingsley C Ekwueme, Nigel J Parr

**Affiliations:** Department of Urology, Wirral University Teaching HospitalWirral, MerseysideUK

## Abstract

**Introduction:**

Urachal cyst is one of a spectrum of urachal abnormalities most commonly found in children. They are very rarely seen in adults because the urachus is normally obliterated in early infancy.

**Case presentation:**

We describe a case of a 32 year old male Caucasian who presented with a tender, midline, infraumbilical mass and purulent umbilical discharge. Diagnosis of an infected urachal cyst was confirmed on magnetic resonance scan. He was treated initially with broad spectrum antibiotics in order to allow sepsis to resolve prior to surgical excision of the cyst and fibrous tract. Cystoscopy was performed intraoperatively to exclude sinus communication with the bladder. Histology of the excised specimen showed chronic inflammation with no evidence of malignancy. Postoperative recovery was uneventful.

**Conclusion:**

Urachal abnormalities are rare in adults. Clinical presentation is non-specific; therefore, a high index of suspicion is required in order to make the diagnosis. When diagnosed, surgical excision is advised because of the risk of malignant transformation.

## Introduction

Urachal abnormalities result from incomplete regression of the foetal urachus. They are more common in children than in adults, due to urachal obliteration in early infancy [1].

In adults, urachal cyst (UC) is the commonest variety, with infection being the usual mode of presentation [2]. Diagnosis remains challenging due to the rarity of this lesion and the non-specific nature of its symptomatology. Since the first description of urachal abnormality by Cabriolus in 1550, few cases have been reported in literature.

We describe a case of urachal cyst presenting with a tender infraumbilical mass, purulent umbilical discharge and sepsis, in a previously fit and well man.

## Case presentation

A 32 year old male Caucasian was referred after presenting to the accident an emergency department with a 10 day history of persistent purulent umbilical discharge associated with constant lower abdominal pain, chills and rigors. He gave no history of nausea, vomiting or change in bowel habit. Systemic review revealed no abnormality. He had completed a course of antibiotics prescribed by his General Practitioner with little relief.

On examination, he was apyrexial and haemodynamically stable. Abdominal examination, however, revealed purulent umbilical discharge with surrounding erythema and a tender infraumbilical mass. A working differential diagnosis of omphalitis or pilonidal disease of the umbilicus, or patent urachus was made.

Haematology showed a raised C-reactive protein of 112 mg/l, but normal white cell count. Microbiology culture of the pus grew *Bacteroides sp*.

**Figure 1.**
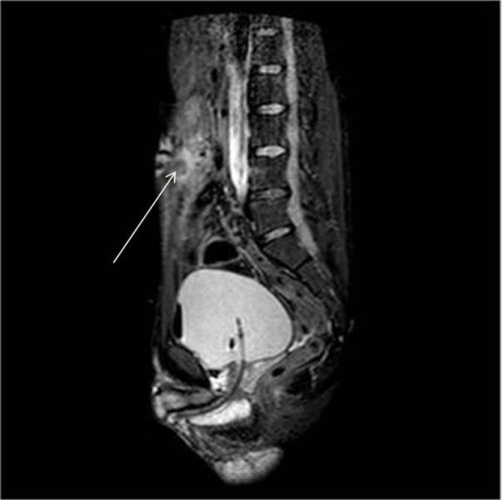
MRI scan showing high signal fluid within the umbilicus tracking into urachal remnant.

Abdominal ultrasound scan showed a 3.8 cm echogenic collection in a cavity within the anterior abdominal wall in the midline. An MRI scan, confirmed the diagnosis of UC communicating proximally with the umbilicus (Figure 1). The initial MRI scan was carried out on an empty bladder making it difficult to exclude possible distal communication between the UC and the bladder. A repeat MRI scan was subsequently performed with a full bladder, to exclude any distal communication.

**Figure 2.**
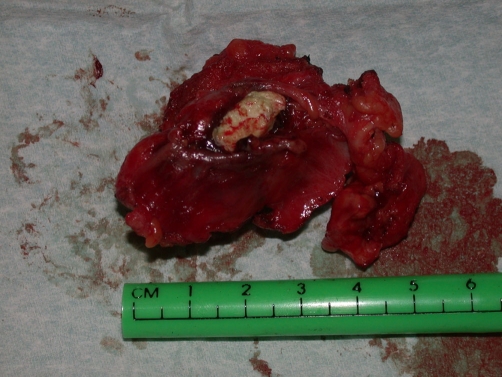
The urachal cyst and fibrous tract containing white cheesy material within its cavity.

The patient was initially treated with intravenous antibiotics followed by a 2 week course of oral antibiotics with surgery planned at the end of this period. Cystoscopy and excision of the infected urachal cyst were performed simultaneously. Cystoscopy confirmed no evidence of a sinus into the bladder. A midline incision was used to excise the cyst, together with the omentum adherent to it posteriorly. The sac of the cyst contained white cheesy material (Figure 2). Histopathological analysis of the resected specimen showed chronic inflammation with no evidence of malignancy. Recovery was unremarkable.

## Discussion

The urachus, developmentally is the upper part of the bladder, both of which arise from the ventral part of the cloaca [3]. Descent of the bladder from the 5^th^ month of development into the foetal pelvis pulls the urachus with it resulting in the formation of the urachal canal. The lumen of this canal progressively obliterates during foetal life, with eventual formation of a fibrous tract in early adult life.

At the end of development, the urachus lies between the transversalis fascia anteriorly and the peritoneum posteriorly (space of Retzius), surrounded by loose areolar tissue and attaches the umbilicus to the dome of the bladder. Histologically, it is composed of 3 layers; an innermost layer of modified transitional epithelium similar to the urothelium, the middle layer of fibroconnective tissue and outermost layer of smooth muscle continuous with the detrusor [1,3].

There are five types of urachal abnormalities: 1) patent urachus, in which the entire tubular structure fails to close; 2) urachal cyst, in which both ends of the canal close leaving an open central portion; 3) urachal sinus, which drains proximally into the umbilicus; 4) vesicourachal diverticulum, where the distal communication to the bladder persists; and 5) alternating sinus, which can drain to either bladder or umbilicus.

The incidence of UC in adults is unknown but it is rare. It is more common in men than women [2,4]. In a 31-year review, Risher et al [2] found 12 adults with urachal anomalies, of which 5 were UC. Modes of presentation of urachal anomalies in adults differ from those seen in children. In adults, the commonest variety is urachal cyst, with infection being usual mode of presentation [2,5]. The route of infection is haematogenous, lymphatic, direct or ascending from the bladder. The commonly cultured microorganisms from the cystic fluid include *Escherichia coli*, *Enterococcus faecium, Klebsiella pneumonia, Proteus, Streptococcus viridans* and *Fusobacterium* [4,5]. In our case, *Bacteroides sp* was cultured.

The clinical signs and symptoms are non-specific, as UC are largely asymptomatic until they become infected. However, the presence of the triad of symptoms including a tender midline infraumbilical mass, umbilical discharge and sepsis should arouse suspicion of UC. If left untreated, UC slowly enlarges and may drain through the umbilicus as was seen in our patient, or drain into the bladder or both, resulting in alternating sinus.

Ultrasound scan can help to make diagnosis in 77% of patients [5]. In our case, ultrasound scan was not specific and MRI scan was used to make diagnosis and define the relationship to surrounding structures.

UC can be complicated by rupture into the peritoneal cavity leading to peritonitis. Other reported complications include uracho-colonic fistula, stone formation and neoplastic transformation [6-9]. The risk of urachal malignancy in adults is high and the prognosis is poor. Ashley et al [4] in a 54 year retrospective study of 130 adults with urachal abnormalities found that 51% were malignant and 20% presented with metastatic disease. The median age at presentation was 61 years and the 2 common risk factors for malignancy were age and haematuria.

Although histologically, the innermost layer of the urachus is mainly transitional cell, adenocarcinoma is the predominant histological type and most are mucinous. This is probably due to metaplasia arising from chronic inflammation.

The treatment of choice for urachal cyst is by complete primary excision. An earlier report suggests a single stage procedure backed with appropriate antibiotic therapy for the treatment of infected UC [10]. However, Yoo et al [5] in their study suggested a 2 stage procedure involving initial incision and drainage, followed by later excision of the urachal remnant. In our case, we adopted a staggered plan of management. Firstly, with administration of broad spectrum antibiotics guided by microbiology sensitivity, and after resolution of sepsis interval primary excision of the cyst, including insertion of a covering corrugated wound drain.

## Conclusion

Urachal anomalies are rare in adults. Presentation is atypical; therefore, a high index of suspicion is required in order to achieve a diagnosis. A triad of lower midline mass, umbilical discharge and sepsis is suggestive, although MRI confirms the diagnosis and defines the surrounding anatomical relationship. Complete surgical excision is the treatment of choice due to the risk of malignant transformation. We recommend a 2 stage treatment with a combination of broad spectrum antibiotics or incision and drainage, followed by interval excision after resolution of sepsis.

## Abbreviation

UC: Urachal cyst
